# Expression of Talin-1 in endometriosis and its possible role in pathogenesis

**DOI:** 10.1186/s12958-021-00725-0

**Published:** 2021-03-09

**Authors:** Xian Tang, Qing Li, Lijie Li, Jianfa Jiang

**Affiliations:** 1 Department of Obstetrics and Gynecology, Loudi Central Hospital of Hunan Province, Loudi, Hunan Province China; 2 Department of Gynecology, The Third Xiangya Hospital, Central South University, NO.138 Tongzipo Road, Yuelu District, Changsha, 410013 Hunan China

**Keywords:** Endometriosis, Talin-1, Adhesion, Migration, Invasion

## Abstract

**Background:**

Endometriosis is a disease that involves active cell invasion and migration. Talin-1 can promote cell invasion, migration and adhension in various cancer cells, but its role in endometriosis has not been investigated. This study was to investigate the expression level of Talin-1 in endometriosis and the role of Talin-1 in the proliferation, adhesion, migration, and invasion of human endometrial stromal cells (ESCs).

**Methods:**

Ectopic and eutopic endometrial tissues were collected from women with endometriosis, and the control endometrial tissues were obtained from patients without endometriosis. The expression level of Talin-1 was detected in each sample using quantitative real-time polymerase chain reaction and immunohistochemistry. The expression of Talin-1 was inhibited using RNA interference in ESCs, and its proliferation, apoptosis, adhesion, migration, and invasion capacity were analyzed. Western blotting was performed to detect the expression of related molecules after the downregulation of Talin-1.

**Results:**

The results showed that the mRNA and protein expression of Talin-1 were significantly increased in the ectopic endometrium and eutopic endometrial tissues compared with the controls. The knockdown of Talin-1 did not affect the proliferation and apoptosis of ESCs. The results indicated that the downexpression of Talin-1 inhibited the adhesion, invasion, and migration of ESCs. In addition, the expressions of N-cadherin, MMP-2, and integrin β3 were significantly lower after the deregulation of Talin-1, whereas the levels of E-cadherin were significantly increased.

**Conclusions:**

The expression of Talin-1 was increased in the ectopic and eutopic endometrial tissues compared with the control endometrium. The downregulation of Talin-1 inhibited the adhesion, invasion, and migration of ESCs.

**Supplementary Information:**

The online version contains supplementary material available at 10.1186/s12958-021-00725-0.

## Background

Endometriosis is a commonly encountered benign gynecologic disease that affects 10% of reproductive-age women [1]. It can be classified into superficial or peritoneal, ovarian, and deep infiltrating endometriosis. It causes chronic pelvic pain, dysmenorrhea, deep dyspareunia, dysuria, dyschezia, fatigue, and infertility, all of which affect physical, mental, sexual, and social well-being as well as productivity [2]. Although endometriosis is a benign disease, endometriotic cells exhibit many features similar to those of malignant cancer, such as migratory and invasive properties. A series of hypotheses that attempt to explain the genesis of endometriosis has been established. However, the etiology and pathogenesis of the disease have not been fully elucidated [3].

The postulated origin of endometriotic tissue is considered retrograde menstruation [4]. However, retrograde menstruation is a very common physiological phenomenon, and only 10% of women develop endometriosis. There must be other factors that promote endometrial cells to adhere to ovaries, ligaments, and peritoneal surfaces and to develop endometriosis. Recent studies have suggested that eutopic endometrium in patients with endometriosis is different from normal endometrial cells, which facilitate the proliferation, implantation, and survival of endometrial tissue in the peritoneal cavity [5]. Recently, the involvement of adhesion molecules in endometriosis has gained much attention. Endometrial stromal cells from women with endometriosis exhibit adhesive capacity as a result of altered integrin profiles. Integrins may promote the attachment of ectopic endometrial cells to the peritoneum [6].

Talin-1, which is located at the adhesion complex between cells and their extracellular matrix (ECM), has been reported to interact with multiple adhesion molecules and to activate integrin and focal adhesion signaling [7]. Recent studies have indicated that the dysregulation of Talin-1 can lead to cell spreading, migration, and survival, and this has led to an extensive investigation into its role in cancer and other disorders [8–12]. Endometriosis is also a disease with active cell migration and invasion. Our previous study demonstrated that the level of Talin-1 was significantly higher in eutopic and ectopic endometrium in women with adenomyosis [13]. However, the relationship between Talin-1 and endometriosis has never been reported. Thus, this study aimed to investigate the expression of Talin-1 in endometriosis and analyzed the potential role of Talin-1 in the development of endometriosis.

## Materials and methods

### Subjects and sample collection

Matched ectopic and eutopic endometrial tissues were obtained from women with ovarian endometriotic cysts undergoing laparoscopic surgery at the Gynecological Department of the Third Xiangya Hospital from January to June 2020. Endometriosis was confirmed through a histological examination. The control endometrial tissues were obtained from patients without endometriosis and women with histologically proven non-endometriotic benign ovarian cysts. All patients had regular menstruation and had not received any hormonal medication within the three months prior to surgery. The samples were collected in proliferating phases of the menstrual cycle, which was determined by preoperative history and histological examination.

### Immunohistochemistry

Immunohistochemistry staining for Talin-1 was performed on 4 μm-thick paraffin sections. Following routine deparaffinization, the sections were hydrated and then microwave-treated for antigen restoration. Endogenous peroxidase activity was eliminated using 3% hydrogen peroxide for 10 min. Then, polyclonal rabbit anti-human Talin-1 (1100 dilution, Bioss, Shanghai, China) was used to incubate overnight at 4 °C. The slides were incubated with a horseradish peroxidase-conjugated antirabbit secondary antibody for 30 min at room temperature. DAB staining was performed. Finally, all sections were counterstained with hematoxylin, dehydrated, and cover-slipped. Images were captured by a Leica DM4000B microscope (Leica, Wetzlar, Germany). The staining intensity was graded as follows: 0 = none, 1 = weak, 2 = moderate, and 3 = strong staining. The percentage of positive stained cells was graded as follows: 0 for no positive staining cells, 1 for 25% positive staining cells, 2 for > 25% and < 50%, and 3 for > 50%. The immunoreactive score was calculated using the following equation: immunoreactive score = staining intensity multiplied percentage of positive cells.

### Western blotting

Western blot analyses were performed as described previously [14]. Briefly, protein was extracted from cells that were lysed in radioimmunoprecipitation buffer and centrifuged at 12,000×g for 15 min at 4 °C. The supernatant protein was quantified by bicinchoninic acid assay (Thermo Fisher Scientific, Rockford, USA). Homogenate proteins were separated by 10% SDS-polyacrylamide gels and transferred onto polyvinylidene difluoride membranes (Millipore, Billerica, MA, USA). The membranes were blocked with 5% fat-free milk, washed, and then probed with the following primary antibodies: β-actin (1:5000 dilution), integrin β3 (1:1000 dilution), N-cadherin (1:2000 dilution), MMP-2 (1:1000 dilution), E-cadherin (15,000 dilution), and Talin-1 (1500 dilution) (all from Proteintech, Chicago, USA). After washing, the membranes were incubated with horseradish peroxidase-coupled goat anti-mouse secondary antibody at room temperature for 80 min. Band intensity was quantified using Quantity One software. β-actin served as the loading control.

### Quantitative real-time polymerase chain reaction (qRT-PCR)

QRT-PCR was performed as previously described [13]. The total RNA was extracted using Trizol reagent (Life Technologies, CA, USA). Reverse transcription was conducted using SuperScript III Transcript (Life Technologies, CA, USA) in accordance with the manufacturer’s protocol. Reactions were performed using the 7500 Real-Time PCR System (Applied Biosystems Inc., Foster City, CA, USA). The primer sequences of Talin-1 and beta-actin are listed in Supplementary Table 1. The relative gene expression of Talin-1 was calculated using the 2^−△△Ct^ method.

### Cell isolation and culture

Endometriotic stromal cells (ESCs) from the eutopic endometrial samples of women with endometriosis culture were processed as described previously [14, 15]. In brief, the samples were collected under sterile conditions, washed, and transferred to the laboratory on ice. Following isolation, the ESCs were passed by using the standard method of trypsinization, plated in culture dishes, and resuspended in phenol red-free Dulbecco’s Modified Eagle’s Medium (DMEM; Gibco; Thermo Fisher Scientific, Inc., Waltham, MA, USA) supplemented with 10% fetal bovine serum (FBS; Sigma-Aldrich, St. Louis, MO, USA) at 37 °C in a humidified atmosphere with 5% CO_2_. The primary ESCs were examined by immunostaining for anti-vimentin (Abcam, Cambridge, MA, USA), a specific marker of stromal cells. Only cultures with more than 96% purity were included in our study.

### Transfection experiments

RNA interference was performed by small interfering RNA (siRNA) transfection. Three different siRNAs targeting Talin-1 and the negative control siRNA were synthesized in GenePharma (Changsha, China). The sequences are listed in Supplementary Table 1. A quantity of10^5^ ESCs was seeded in a six-well plate the day before transfection. The transfection of the control siRNA or siRNA against Talin-1 was conducted using Lipofectamine 2000 (Invitrogen, Carlsbad, CA, USA) according to the manufacturer’s protocol. After 24 h, the cells were harvested for Western blot to confirm the gene silence. The cells were harvested after 48 h for other assays.

### Cell proliferation assay

The proliferation of ESCs was determined using Cell Counting Kit (CCK)-8 assays. After being transfected with siRNAs, the ESCs were seeded in 96-well plates at a density of 1 × 10^4^ cells in 100 μL of culture medium per well. After a culture time of 0, 24, 36, and 48 h, 20 μL of CCK-8 (Dojindo, Kyushu, Japan) was added for an additional 4 h of incubation. A spectrophotometric plate reader (Thermo Fisher Scientific, Inc., MA, USA) was used to read the absorbance at 450 nm. This experiment was performed with triplicate wells and independently repeated at least three times.

### Apoptosis assay by flow cytometry

Apoptotic cell death was measured by a fluorescein isothiocyanate-annexin V/PI apoptosis detection kit (KeyGEN BioTECH, Nanjing, China) according to the manufacturer’s protocol after transfection with siRNAs for 48 h. The cells were washed twice with phosphate buffered saline (PBS) and resuspended in a binding buffer at a concentration of 1 × 10^5^ cells/mL. The cells were stained with annexin V-APC and propidium iodide and detected by flow cytometry. The cells labelled with annexin V were considered apoptotic, and propidium iodide was used to detect dead cells. Fluorescence-activated cells were quantified using a flow cytometer. The apoptotic rate was the sum of annexin V-positive/ propidium iodide-negative and annexin V-positive/propidium iodide-positive cells in two quadrants as a proportion of the total number of cells.

### Adhesion assay

A CCK-8-based adhesion assay was used to determine the adhesion capacity of the ESCs. A 96-well plate was used to perform this assay. Matrigel of 50 μL in a serum-free medium (at 1:8 dilution) was added to each well. After transfection for 48 h, 1 × 10^4^ cells/100 μL were cultured in each incubated well. After incubation for 30 min, the non-adherent cells were rinsed off, and 10 μL CCK-8 (Dojindo, Kyushu, Japan) was added for an additional 4 h of incubation. A spectrophotometric plate reader was used to read the absorbance at 450 nm. The number of adhesion cells was characterized by the OD value.

### Wound-healing assay

A wound-healing assay was performed to evaluate the cells’ ability to migrate. Briefly, the cells transfected with Talin-1 siRNA were seeded into six-well culture plates at 90% confluency. A wound was made on a monolayer of cells using a standard 1000 μL plastic pipette tip. The cells were washed using PBS to clear away the cell debris. For each well, pictures were taken at 0, 24, and 48 h after the wound. The wound width was calculated using Image-Pro Plu software. The migration rate was calculated as [Cell-free area (0 h)- Cell-free area (24 h or 48 h)]/Cell-free area (0 h).

### Transwell invasion assay

Transwell invasion assay was performed to assess the invasion of the ESCs. In the invasion assay, the upper chamber was first coated with 60 μL matrigel (1:2 matrigel and DMEM without phenol red) and incubated for 1 h at 37 °C. The ESCs (10^5^ cells/well) were plated into the upper chambers, and the lower chambers were filled with phenol red-free DMEM plus 10% FBS. After 72 h of incubation, the cells in the upper chamber were removed, and the transwell filters were fixed with 4% paraformaldehyde for 30 min, washed with PBS twice, stained with 0.5% hematoxylin for 5 min, and counted in three representative fields under a light microscope (Olympus). A spectrophotometric plate reader was used to read the absorbance at 550 nm. The experiments were performed in triplicate.

### Statistical analysis

All statistical analyses were performed using SPSS software version 22.0 (SPSS, Inc., Chicago, USA). All data are presented as means ± standard deviation. One-way analysis of variance was used to analyze the differences among multiple groups. *P* values < 0.05 was considered statistically significant.

## Results

### Increased Talin-1 protein expression in endometriosis

Ectopic and eutopic endometrium tissues were obtained from 26 patients with endometriosis (mean age 36.65 ± 6.99 years), of whom 15 had AFS stage III disease and 11 had stage IV disease. Samples of the control endometrium were obtained from 15 women without endometriosis (mean age 35.53 ± 5.40 years).

**Fig. 1:**
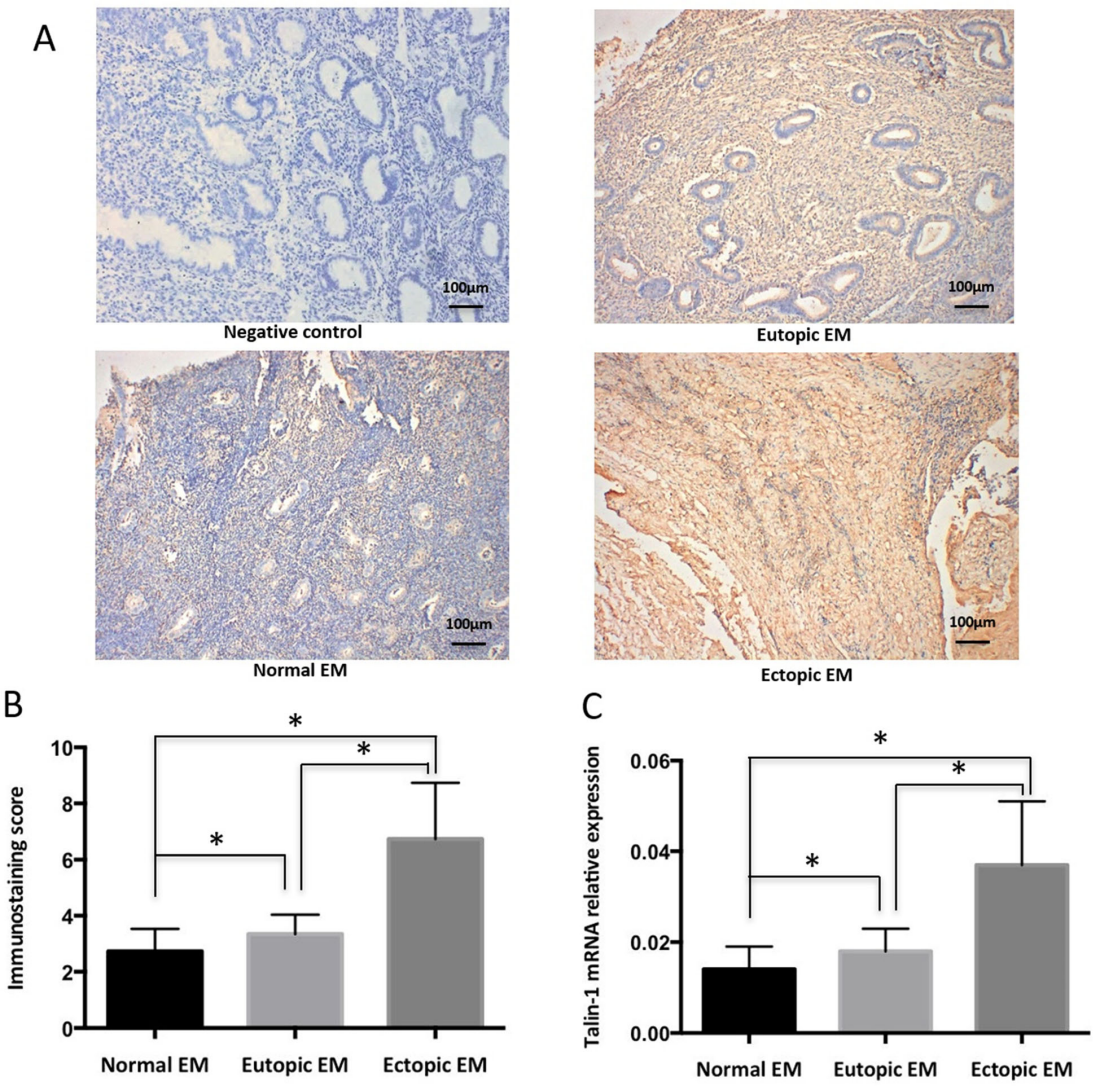
The mRNA and protein levels of Talin-1 were upregulated in human endometriotic tissues. **a** Representative images of different tissues showing the Talin-1 immunohistochemical staining. The images of eutopic EM and ectopic EM were from the same patient. The original magnification was 100×. **b** Immunostaining score analysis of talin1 protein expression in different tissues. **c** QRT-PCR analysis of talin1 mRNA expression in human endometriotic tissues. *P<0.05

Immunohistochemical staining of the tissue showed that Talin-1 was present in both epithelial and stromal cells, and that the staining was mostly cytoplasmic (Fig. 1a). Compared with that in the normal endometrium (2.73 ± 0.80), the Talin-1 protein expression in the eutopic (3.35 ± 0.69) and ectopic endometrium (6.73 ± 2.01) of ovarian endometriosis was significantly increased (*P* = 0.014; *P* < 0.001, respectively). The protein levels of Talin-1 in the ectopic endometria was significantly increased compared with the eutopic levels (*P* < 0.001) (Fig. 1b).

### Increased Talin-1 mRNA expression in endometriosis

Compared with those in the control group (0.014 ± 0.005), the mRNA levels of Talin-1 in the ectopic endometrium (0.037 ± 0.014) and eutopic endometrium tissues (0.018 ± 0.005) of patients were significantly higher (*P* < 0.001 and *P* = 0.013, respectively) (Fig. 1c). When tests were performed on the matched samples of eutopic and ectopic endometrium tissues of women with endometriosis, the expression of Talin-1 was significantly increased in the ectopic endometrium (*P* < 0.001).

### Silencing of Talin-1 had no effect on the proliferation and apoptosis of ESCs

**Fig. 2:**
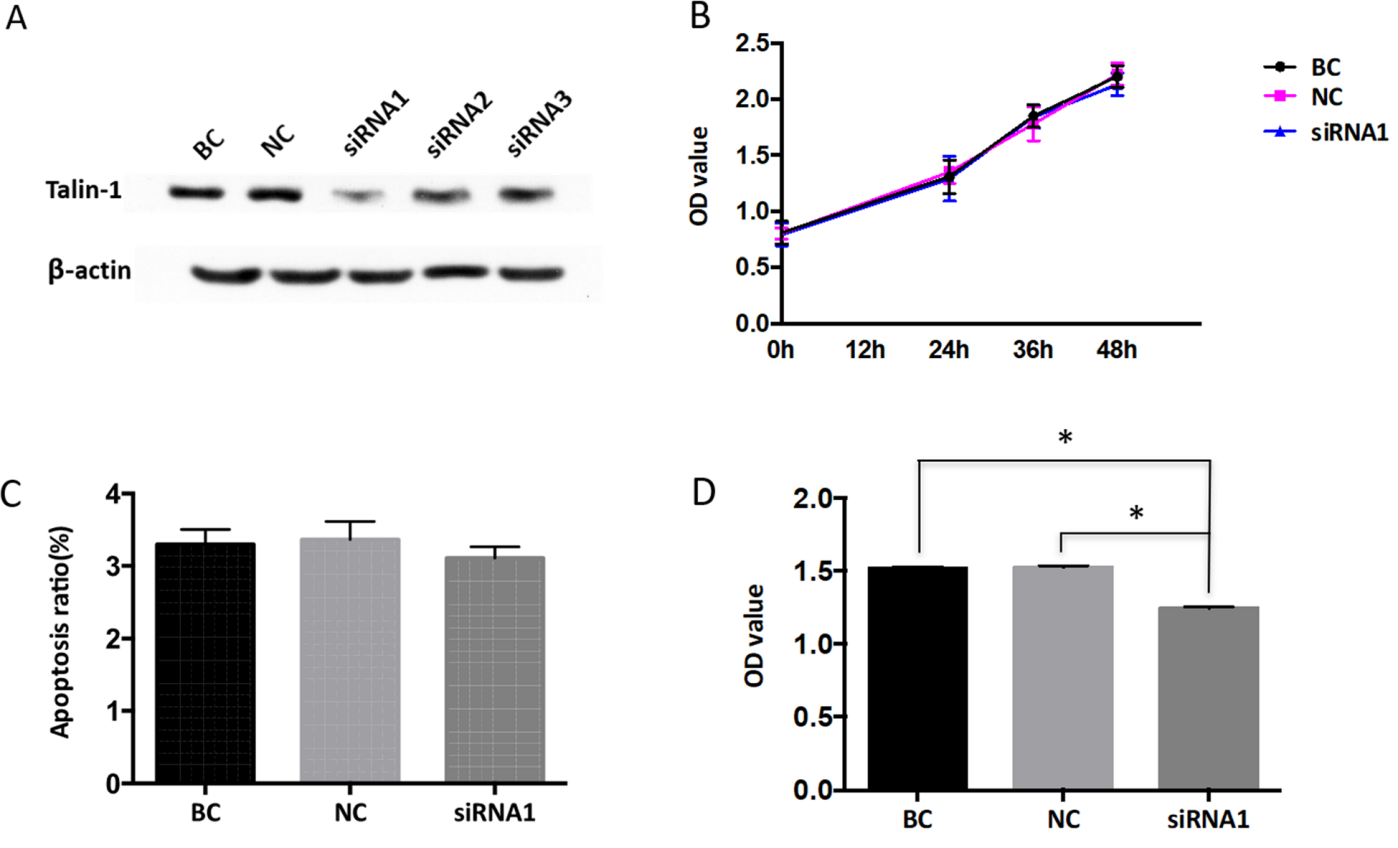
The effect of Talin-1 on the proliferation, apoptosis and adhension capacity of ESCs. **a** Down-regulation of Talin-1 assessed by western blotting after transfection with three short interfering RNA (siRNA) or the negative control (NC). The first siRNA (siRNA1) was selected for further investigations. BC, blank control without siRNA. **b** Knockdown of Talin-1 had no effect on the proliferation of ESCs. **c** Knockdown of Talin-1 had no effect on the apoptosis of ESCs. **d** Down-regulation of Talin-1 inhibited the adhesion of ESCs. *P<0.05

As the Talin-1 level was upregulated in human endometriotic tissues, we knocked down its expression in the ESCs to investigate its functional roles. The ESCs were transfected with three siRNA sequences targeting Talin-1. Western blot analysis showed that the protein expression of Talin-1 was significantly decreased in the si-Talin-1 group compared with the negative control (NC) group and blank control (BC) group (Fig. 2a). According to the results of the Western blot analysis, the first siRNA (siRNA1) was selected for further investigations.

To determine the effect of Talin-1 on the proliferation and apoptosis of the ESCs, the cells were transfected with siRNA1 or si-NC for 48 h, followed by CCK-8 and flow cytometry. The CCK-8 assays revealed that the silencing of Talin-1 did not affect the proliferation of the ESCs compared with the NC group and BC group (Fig. 2b). Flow cytometry showed that the downregulation of Talin-1 did not promote the apoptosis of the ESCs compared with the controls (Fig. 2c).

### Talin-1 knockdown inhibited the adhesion of ESCs

The effect of Talin-1 knockdown on the adhesion ability of the ESCs was examined in vitro. In the adhesion assay, the downregulation of Talin-1 effectively suppressed the adhesion of the ESCs (Fig. 2d) compared with the control cells.

### Downregulation of Talin-1 inhibited the migration and invasion of ESCs

**Fig. 3:**
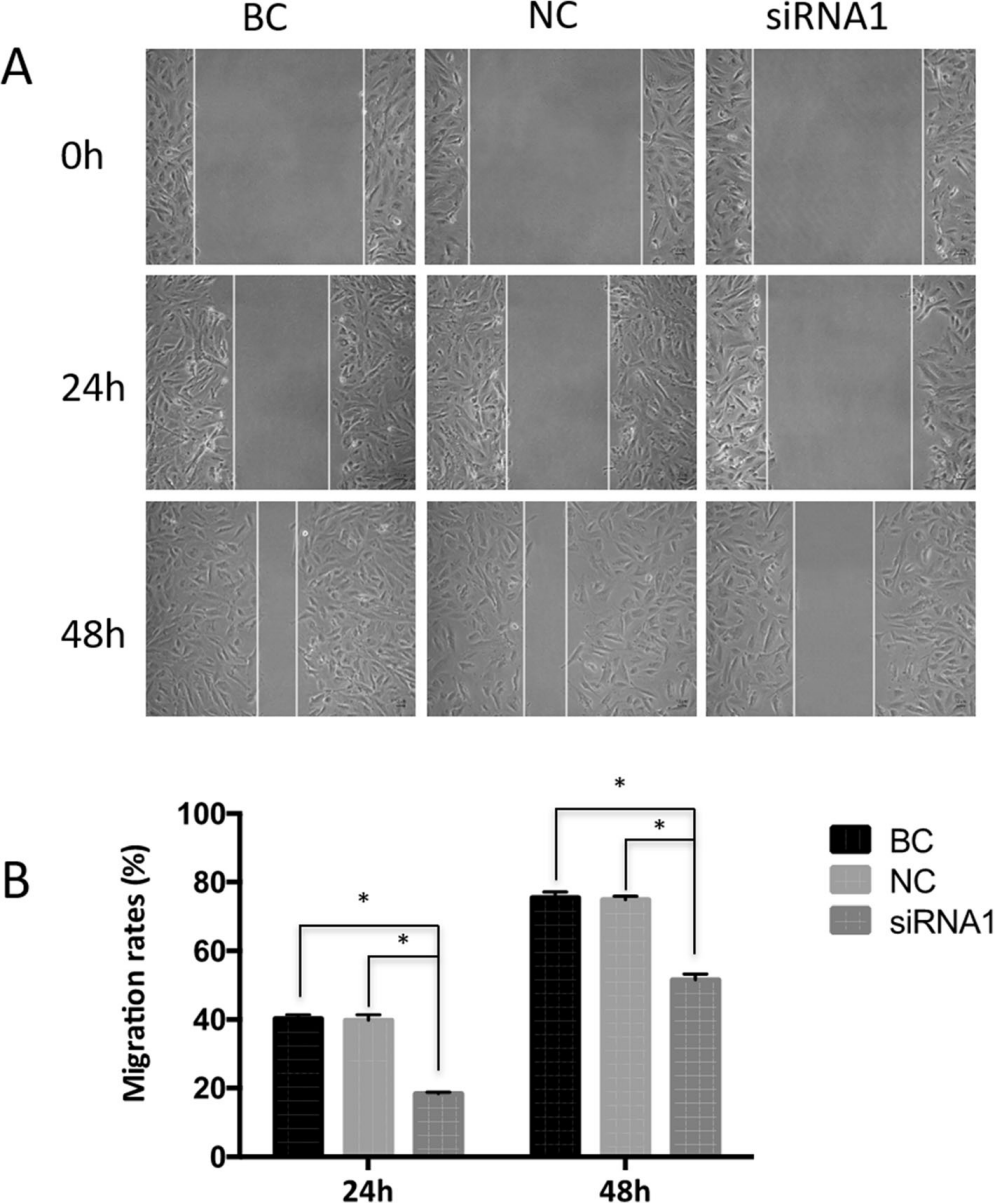
Downregulation of Talin-1 inhibited the migration of ESCs **a** Migration of ESCs transfected with siRNA1 was assessed in wound healing assays. BC, blank control without siRNA. NC, negative control. Magnification, 400×. **b** Quantification results of the wound-healing assays (mean ± SD). *P<0.05

To investigate the effects of Talin-1 downexpression on ESC migration, wound-healing assay was performed. As shown in Fig. 3, the silencing of Talin-1 led to a significant decrease in ESC migration ability compared with that in the NC group and BC group.

### Talin-1 knockdown inhibited the invasion of ESCs

**Fig. 4:**
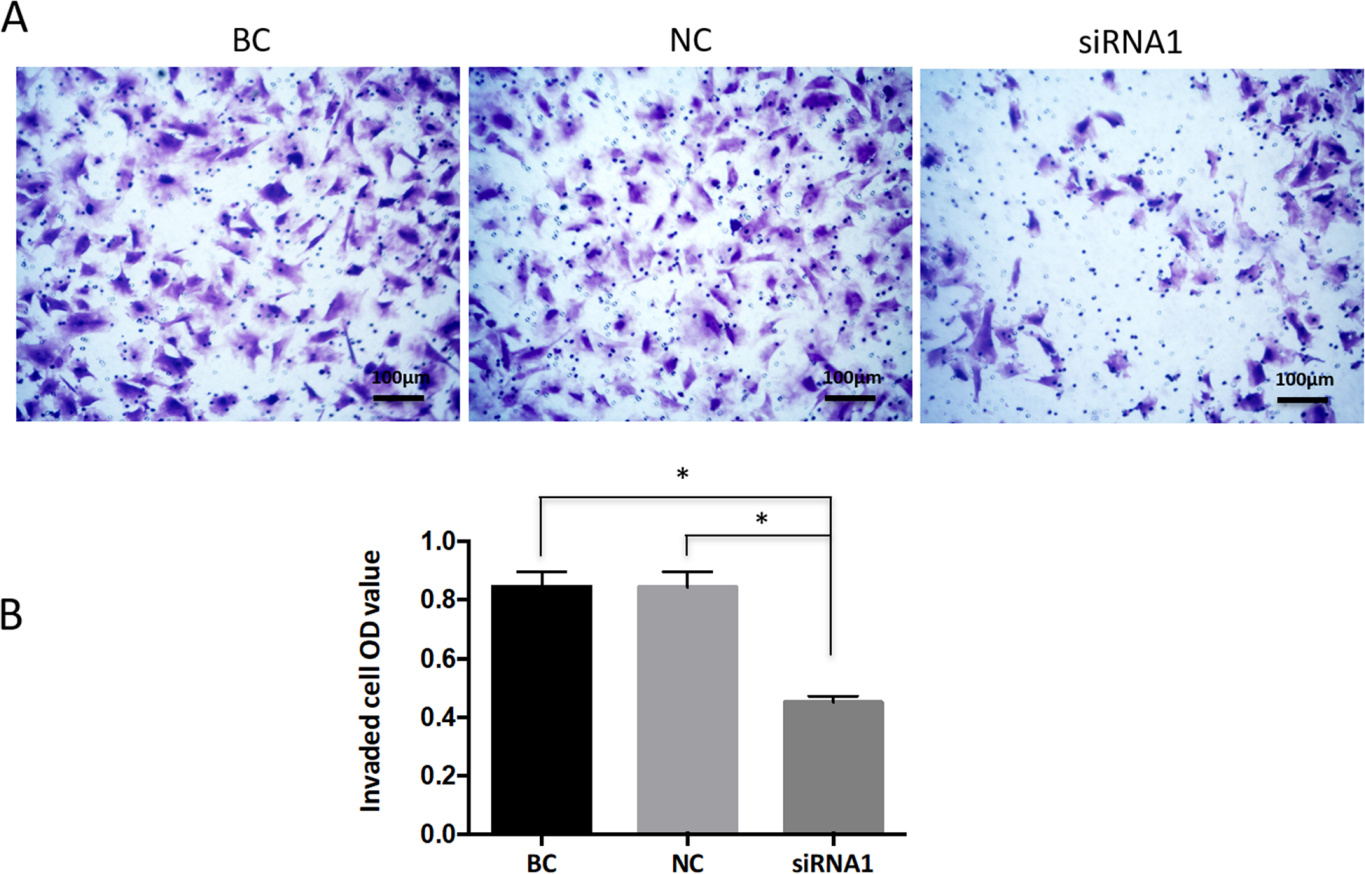
Downregulation of Talin-1 inhibited the invasion of ESCs. **a** Invasion of ESCs transfected with siRNA1 was assessed in transwell invasion assays. BC, blank control without siRNA. NC, negative control. Magnification, 400×. **b** Quantification results of the transwell invasion assays (mean ± SD). *P<0.05

The relationship between Talin-1 knockdown and cell invasion was analyzed using siRNA technology in the ESCs. The results showed that the number of ESCs that had invaded through the Matrigel-precoated transwell filters was significantly reduced in the siRNA1 group (Fig. 4). The negative control siRNA did not significantly interfere with the invasion ability of the ESCs compared with the BC group.

### Effects of Talin-1 on the related molecule expression in ESCs

**Fig. 5:**
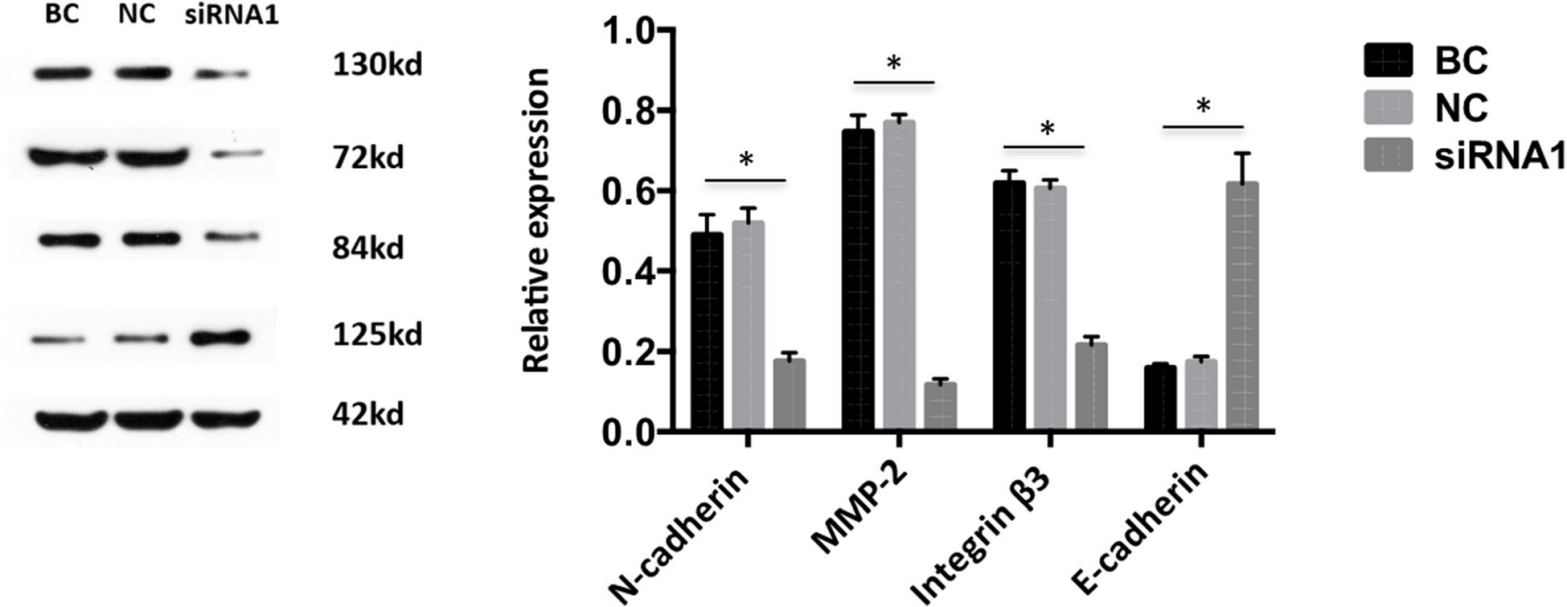
Effects of Talin-1 down-regulation on related molecule expression. **a** Representative western blots analysis, with values normalized to β-actin. **b** Quantification results of Talin-1 down-regulation on related molecule expression. The results showed that down-regulation of Talin-1 obviously decreased the expression of integrin β3, N-cadherin, MMP-2 in ESCs compared to BC group and NC group, while the expression of E-cadherin was increased. *P<0.05

The effect of Talin-1 on the expressions of adhesion-related molecule integrin β3, migration-related molecule N-cadherin and E-cadherin, and invasion-related molecule MMP-2 on ESCs was assessed using Western blot after transfection with siRNA1 for 48 h. The results showed that the downregulation of Talin-1 clearly decreased the expression of integrin β3, N-cadherin, and MMP-2 in the ESCs compared with the BC group and NC group, whereas the expression of E-cadherin increased (Fig. 5).

## Discussion

To the best of our knowledge, this study is the first report on the expression of Talin-1 in endometriosis and its possible role in pathogenesis. We identified that the mRNA and protein of Talin-1 were highly expressed in the ectopic and eutopic endometrial tissues of patients with endometriosis compared to the control endometrium tissues. The downregulation of the expression of Talin-1 in the ESCs showed decreased adhesion, migration, and invasion ability, confirming the involvement of Talin-1 in the pathological endometriotic process. The knockdown of Talin-1 affected the expression of the adhesion-related molecule integrin β3, migration-related molecules E-cadherin and N-cadherin, and invasion-related molecule MMP-2. The present findings provide novel insights into the role of Talin-1 in endometriosis.

Talin-1 is a focal adhesion protein that binds to multiple adhesion molecules and is an essential mediator of cell–ECM adhesion [16, 17]. The N-terminal head domain of Talin-1 binds to the integrin β subunit cytoplasmic domain, which causes integrin activation and stimulates integrin binding to the ECM [18, 19]. Moreover, Talin-1 can act independent of integrins by suppressing the expression of E-cadherin, which is a cell–cell adhesion molecule [20]. Accumulating evidence suggests that the expression of Talin-1 is dysregulated in various malignant neoplasms, such as colorectal cancer [21], hepatocellular carcinoma [22], prostate cancer [16], and oral squamous cell carcinoma [23]. Although endometriosis is a benign disease, it exhibits many cancer-like features, such as proliferation, anti-apoptosis, and cell migration. Whether Talin-1 is associated with the pathophysiology of endometriosis remains unclear. In the current study, we found that Talin-1 expression at both mRNA and protein levels was significantly upregulated in the eutopic and ectopic endometrial tissues of endometriosis compared with the controls, suggesting that Talin-1 could be associated with the genesis and progress of endometriosis. But it’s worth noting that the increase in the expression of Talin-1 in the ectopic endometrial tissues of patients with endometriosis was higher than that in eutopic endometrium. The specific reason is unclear, which may be related to stronger adhesive behavior of ectopic endometrial stromal cells [24].

However, the role of Talin-1 in endometriosis is not clear. Some studies have indicated that endometriotic cells have aggressive ability. Talin-1 has been described as an oncogene, and it mediates cell adhesion, proliferation, tumorigenesis, and metastasis. Talin-1 overexpression markedly enhanced the migration and invasion potential of human prostate cancer cells by activating ECM–integrin-mediated signaling and promoting anoikis resistance [16]. Talin-1 can also significantly promote hepatocellular carcinoma cell proliferation and metastasis. Integrin signaling has been shown to be crucial in cell invasion and migration not only by physically tethering cells to the matrix but also by sending and receiving molecular signals [25]. Based on the above findings, the high expression of Talin-1 could be associated with endometrial tissue adherence and migration at ectopic sites to form endometriotic lesions. In the current study, we found that the downregulation of Talin-1 could inhibit the adhesion, migration, and invasion of endometrial stromal cells.

Talin-1 regulates integrin and focal adhesion signaling. A recent study showed that Talin-1 played an important role in integrin activation, cell adhesion, migration, invasion, and anoikis of prostate cancer cells, and promotion of prostate cancer bone metastasis [12]. At the initial stages of endometriosis, the attachment of retrograde endometrial tissues onto the pelvic mesothelium is a critical step [26]. Several integrins, including αv, β3, β4, and β1, have been reported to mediate the attachment of endometrial cells to the mesothelium [27, 28]. The expression of these integrins is tightly regulated by diverse molecules. The results of the current study showed that the knockdown of Talin-1 affected the expression of integrin β3, indicating that Talin-1 could promote adhesion and migration by regulating integrin β3.

The pathogenesis of endometriosis also requires ECM breakdown. The involvement of MMPs in the development of endometriosis has been confirmed. MMP-2 is one of the members of the MMP family proteins that play an important role in the formation of endometriosis. It can degrade ECM and increase activity in endometriosis as well as mediate the migration and invasion of endometriotic cells [29, 30]. The current study showed that the expression of MMP-2 was positively related with Talin-1, suggesting that Talin-1 could be involved in the invasion process by regulating MMP-2 expression. However, the underlying mechanism remains to be clarified.

This study has several limitations, including lacking ESCs from women without endometriosis in parallel, lack of an established physiologic level of Talin-1 in ESCs, lacking ectopic ESCs experiments, and the possibility of off-target effects from siRNAs. We plan to conduct further studies to determine which parts of Talin-1 protein are responsible for the observed effects. We also want to know what is downstream of Talin-1 and how does it relate to integrins. In the further studies, we will include ESCs from women without endometriosis and ectopic ESCs from patients with endometriosis in parallel to firmly establish our findings.

## Conclusion

We showed that the expression of Talin-1 was elevated in women with endometriosis. The knockdown of Talin-1 could decrease cell adhesion, migration, and invasion in eutopic endometriotic stromal cells from women with ovarian endometrioma. These effects were mediated by regulating MMP-2 and integrin β3. Our findings can provide insights into the possible role of Talin-1 in the genesis and progress of endometriosis.

## Supplementary Information

**Additional file 1: Supplementary Table 1.** The sequences of Talin-1 and siRNA targeted to Talin-1.

## Data Availability

The datasets used and/or analyzed during the current study are available from the corresponding author on reasonable request.

## Abbreviations

ESCs: Endometrial stromal cells
ECM: Extracellular matrix
qRT-PCR: Quantitative real-time polymerase chain reaction
siRNA: Small interfering RNA
PBS: Phosphate buffered saline
CCK: Cell Counting Kit
NC: Negative control
BC: Blank control
