# Supplementary material for: Expression of Talin-1 in endometriosis and its possible role in pathogenesis
Source: Reprod Biol Endocrinol. 2021 Mar 9;19:42. doi: 10.1186/s12958-021-00725-0 (PMC7942010; doi:10.1186/s12958-021-00725-0)
Supplement: Supplementary file 1 — Additional file 1: Supplementary Table 1. The sequences of Talin-1 and siRNA targeted to Talin-1. [file 12958_2021_725_MOESM1_ESM.docx]

Supplementary Table 1 The sequences of Talin-1 and siRNA targeted to Talin-1

| Gene | Forward | Reverse |
| --- | --- | --- |
| Talin-1 | 5'-GGACACATGCCTCCTCTGACT-3' | 5'-CAGGCCTTAGAGGCAGCATC- 3' |
| siRNA1 | 5'-GGCUCUUUCUGUCAGAUGATT-3' | 5'-UCAUCUGACAGAAAGAGCCTT-3' |
| siRNA2 | 5'-CAGGGACCUUAAGAAAGGATT-3' | 5'-UCCUUUCUUAAGGUCCCUGTT-3' |
| siRNA3 | 5'-GAGAUGAGGAGUCUACUAUTT-3' | 5'-AUAUUAGACUCCUCAUCUCTT-3' |
| siRNA NC | 5'-UUCUCCGAACGUGUCACGUTT-3' | 5'-ACGUGACACGUUCGGAGAATT-3' |
| β-actin | 5'-GCCGTGGTGGTGAAGCTGT-3' | 5'-ACCCACACTGTGCCCATCTA-3' |
